# Cultural background, gender, and institutional status have an effect on the evaluation of multi-disciplinary participatory action research

**DOI:** 10.1371/journal.pone.0196790

**Published:** 2018-05-04

**Authors:** Frieder Graef, Stefan Sieber

**Affiliations:** Leibniz Centre for Agricultural Landscape Research (ZALF), Müncheberg, Germany; TNO, NETHERLANDS

## Abstract

Research and development increasingly apply participatory approaches that involve both stakeholders and scientists. This article presents an evaluation of German and Tanzanian researchers’ perceptions during their activities as part of a large interdisciplinary research project in Tanzania. The project focused on prioritizing and implementing food-securing upgrading strategies across the components of rural food value chains. The participants involved during the course of the project were asked to provide feedback on 10 different research steps and to evaluate eight core features related to the functioning and potential shortcomings of the project. The study discriminated among evaluation differences linked to culture, gender, and institutional status. Perceptions differed between Tanzanian and German participants depending on the type and complexity of the participatory research steps undertaken and the intensity of stakeholder participation. There were differences in perception linked to gender and hierarchical status; however, those differences were not as concise and significant as those linked to nationality. These findings indicate that participatory action research of this nature requires more targeted strategies and planning tailored to the type of activity. Such planning would result in more efficient and satisfactory communication, close collaboration, and mutual feedback to avoid conflicts and other problems. We further conclude that it would be advisable to carefully incorporate training on these aspects into future project designs.

## Introduction

As a result of on-going and anticipated regional food crises, regional and global partnerships focused on research and development have been motivated to upgrade local and regional food systems and develop region-specific and innovative strategies [1,2]. Recent research and development (R&D) projects have increasingly focused on using stakeholder-oriented participatory approaches to upgrade entire food systems and increase food security [3]. In these projects, stakeholders are considered to be co-generators of knowledge in a transdisciplinary setting [4]. Because stakeholders’ ability to change their social and economic circumstances is often constrained by various forms of social, cultural and political domination [5,6], participatory approaches consider specific cultural, political, social, ecological and economic environments [7] and aim to ensure the broad participation of local and regional stakeholders [8,9].

Large multi-disciplinary and participatory R&D projects, however, need to tackle diverse topics, activities and staff requirements. These demands entail increased transaction costs [10,11] and require highly complex research management structures for decision-making and communication [12]. This requirement applies in particular to virtual multicultural project teams, where face-to-face meetings are unlikely or rare. It also applies to a north-south context with mixed teams, where cultural, gender and status-related perceptions are likely to differ [5,13,14]. Ultimately, these differences affect communication and team relations [15]. [10] Investigations of communication effectiveness within multicultural project environments have found several determining factors, such as awareness of cultural variation, development of effective trust, communication skills and empathy. However, such investigations have also indicated the need for future research on strategies for effective collectivism and communication in multicultural project teams.

Increased transaction costs and various activity and communication drawbacks were recently experienced in a large interdisciplinary participatory action research (PAR) project in Tanzania. In this project, 10 upgrading strategies (UPS) for enhancing food value chains (FVC) were selected and implemented through a participatory process [16] involving both local subsistence farmers and agricultural scientists. This multicultural project [17] consisted of more than 120 scientists and non-scientists of different nationalities from 16 institutes; these individuals worked mostly as virtual teams in Germany and Tanzania. The activity and communication drawbacks experienced during this project resulted in considerations of their possible causes and remedies.

The authors, therefore, investigated the perceptions of various participatory project activities and steps held by different types of scientists involved during three project years; the scientists were distinguished by their nationality/culture, gender and institutional status. The objective of this study was to examine the effect of these three factors on perceptions of specific activities as well as on the type of collaboration required. This would contribute to the understanding of the shortcomings and requirements of this type of large intercultural R&D project, with the ultimate aim of enhancing PAR communication in different cultural and rural settings [18,19].

## Methodology

### Definitions of key terms

Participatory action research (PAR): Core features of PAR are “its orientation towards taking action, its reflexivity, the significance of its impacts and that it evolves from partnership and participation.” Bradbury-Huang [20]. Research methodologies and activities are context-oriented and iterative with regards to how participants select methods, collect data, and reflect in cycles on how change and the impact of the intervention unfold [4].

Upgrading strategies (UPS): In this context, this term is used for sets of activities and/or technologies among the FVC components, which improve food security at the village level.

Food value chain (FVC): A FVC is defined as consisting of the following main components: natural resources for food production, primary production, food processing, marketing, and consumption. In this, project the main food commodities were maize, millet, groundnut, sunflower, and pigeon pea.

### Organisational structure of the PAR project

The large interdisciplinary PAR project through which we investigated members’ perceptions was coordinated in both Germany and Tanzania. The scientific and management responsibility and budget, as well as the participatory FVC research process, were controlled in Germany, while many research activities were decentrally organized, with each partner having their own research activity and budgetary responsibilities. The 600 local stakeholders involved in implementing, testing and assessing the UPS were primarily coordinated and managed by scientific and non-scientific Tanzanian partners. More than 120 scientists and non-scientists were involved, and they came from 14 scientific institutions: seven German universities and research centres; five Tanzanian universities, research centres and NGOs; and two international research centres from Kenya and the US. Potential coordination problems and interpersonal collaboration conflicts were managed by either the coordinators or a conflict management unit [21].

### Participatory action research steps used for subsequent evaluation

At the start of the project, a roadmap was drafted that depicted the likely participatory process and sequence of steps [16,22]. Local stakeholder and scientist interaction was an integral part of most project activities and served to co-generate knowledge at different levels of intensity [20], iteratively shaping most of the participatory research steps as described below. Some of these steps were conceptualised to be unique and to depend on input-output relations, while other more reflective PAR steps, such as monitoring and impact assessment, were iterative and resulted in gradual adaptation of the PAR. These adaptations required higher levels of north-south and scientist-to-stakeholder communication [23].

The participatory research steps in this project are described as follows:

1. *Mapping stakeholders across the FVC*, including all relevant key and grassroots-level stakeholders and their functions at local, regional, and national scales.
2. *Inventorying FVC constraints & strategies* across priority commodities among rural farmers.
3. *Identifying local food security criteria* for assessing the impact of UPS. This was done using existing literature, many local focus groups, and panel discussions.
4. *Identifying three to five UPS per FVC component* using fact sheets and an inventory established for the case study sites, the target regions, and beyond. These were discussed in depth among scientists with regards to expected positive impacts on food and livelihood security as well as other factors.
5. *Prioritizing UPS* in case study sites *for testing* by stakeholder groups at all case study sites.
6. *UPS group formation* of a total of 24 farmer groups with sizes ranging from 10 to 50 members.
7. *UPS implementation*, *testing*, *and adaptation* at the case study sites with recurrent feedback and adaptation activities between local stakeholders and scientists extending over several months up to two years.
8. *Co-creation of potential future scenarios* with researchers to prove implemented UPS against changing frame conditions.
9. *UPS monitoring & impact assessment* by using generic and specific parameters collected during both UPS focus group discussions and visits to all involved households.
10. *UPS out- and up-scaling* of lessons learnt via the research network, stakeholder organizations and capacity-building workshops at the policy, extension and farmer-school levels.

**Fig 1:**
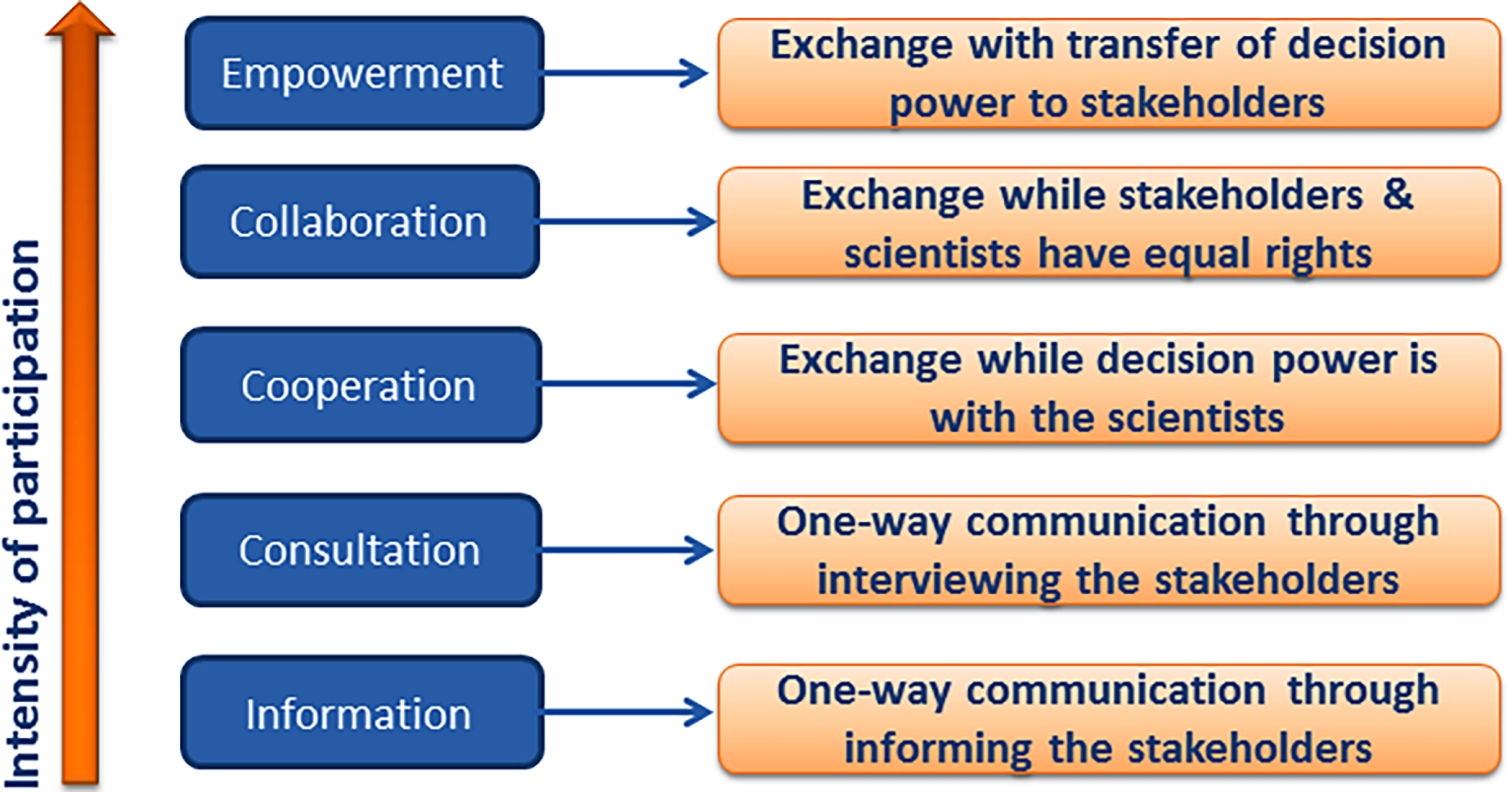
Intensity levels of participation [23, modified].

The levels of participation intensity (Fig 1) applied across all participatory research steps are indicated in Tables 1 and 2. They spanned from mere information (low participation intensity) to entire stakeholder empowerment (high participation intensity).

**Table 1:** Assessment of selected analytical steps undertaken across participatory research actions (Steps (1) Mapping stakeholders across FVC; (2) Inventorying FVC constraints & strategies; (3) Identifying food security criteria; (4) Identifying 3–5 UPS per FVC component; (5) Prioritising UPS in CSS for testing; (6) UPS groups formation; (7) UPS implementation, testing, adaptation; (8) Creation of potential future scenarios; (9) UPS monitoring & impact assessment; (10) UPS out and up-scaling.

Guidance/instructions provided by coordinators		Step 1			Step 2			Step 3			Step 4			Step 5		
No. of involved scientists		9			8			10			22			14		
No. of involved stakeholders		120			80			120			0			200		
Stakeholder participation intensity levels^1^		1			1			0;1;2			0			0;1;4		
Amount of instructions provided by coordination or team leaders (0–4) ^2^		1			2			1			4			3		
Degree of cooperation between WP & Tasks (No. activities, institutions) (0–4) ^2^		1			1			3			4			4		
Time period allocated by coordination (No. of days)		45			30			90			60			60		
Delay (No. of days) ^3^		90			45			30			60			10		
No. reminders for finalising actions		5			2			1			15			2		
**Parameters assessed by involved project members**		mean	SD	(N)	mean	SD	(N)	mean	SD	(N)	mean	SD	(N)	mean	SD	(N)
Need for more instructions (0–4) ^2^	G	1,0		1	3,0		1	1,0	1,0	3	2,8	1,5	9	-		1
	TZ	1,2	1,1	5	2,5	1,0	6	3,0	1,4	4	2,9	1,1	9	2,4	1,3	10
Complexity/multidisciplinarity of activity (0–4) ^2^	G	3,0		1	3,0		1	3,7	0,6	3	3,1	1,1	10	4,0		1
	TZ	2,2	1,1	5	2,7	0,8	6	2,8	1,0	4	2,7	0,9	9	2,9	1,0	10
Communication requirements among members during this activity (0–4) ^2^	G	2,0		1	2,0		1	3,7	0,6	3	3,5	0,8	10	3,0		1
	TZ	3,8	0,5	4	3,4	0,5	5	3,3	0,6	3	3,5	0,5	8	3,6	0,7	9
Degree of stakeholder participation (0–4) ^2^	G	3,0		1	4,0		1	3,3	0,6	3	1.9*	1,4	10	4,0		1
	TZ	3,4	0,5	5	3,8	0,4	6	3,8	0,5	4	3.3*	0,9	9	3,6	0,5	10
Percentage of task completed (%)	G	40		1	70		1	100	-	3	85	18,8	9	100		1
	TZ	81	24,7	5	91	3,5	5	92	6,8	3	83	34,6	8	96	6,2	9
Project member satisfaction during process (0–4) ^2^	G	1,0		1	1.5		1	3,3	0,6	3	1.9**	0,9	10	3,0		1
	TZ	3,2	0,4	5	3.0	-	6	3,3	0,5	4	3.1**	0,6	9	3,2	0,4	10
Final project member’s satisfaction after >2 years (0–4) ^2^	G	1,0		1	1,0		1	3,7	0,6	3	2,2	0,8	10	2,0		1
	TZ	3,0	0,8	4	3,2	0,4	5	3,3	0,6	3	3,0	0,9	8	3,2	0,4	9
Amount of conflicts / tension experienced (0–4) ^2^	G	2,0		1	4,0		1	1,3	0,6	3	1,8	1,4	10	1,0		1
	TZ	0,2	0,4	5	0,7	0,8	6	0,3	0,5	4	1,1	0,8	9	1,3	1,2	10

^1^ Stakeholder participation intensity levels: 0: information; 1: consultation; 2: cooperation; 3: collaboration; 4: empowerment (Fig 1)

^2^ ratings: 0: none; 1: low; 2: medium; 3: high; 4: very high

^3^ accumulated No. of days for one step

significance level at

*p < 0.05

**p < 0.01 (Mann-Whitney U-test)

**Table 2:** 

Guidance/instructions provided by coordinators		Step 6			Step 7			Step 8			Step 9			Step 10		
No. of involved scientists		12			28			12			28			55		
No. of involved stakeholders		550			560			-			580			2000		
Stakeholder participation intensity levels^1^		2;3;4			3;4			0;1			1			0;1;2		
Amount of instructions provided by coordination or team leaders (0–4) ^2^		2			3			2			3			2		
Degree of cooperation between WP & Tasks (No. activities, institutions) (0–4) ^2^		2			4			2			4			2		
Time period allocated by coordination (No. of days)		45			150			60			900			750		
Delay (No. of days) ^3^		40			100			60			60			120		
No. reminders for finalising actions		2			20			5			8			6		
**Parameters assessed by involved project members**		mean	SD	(N)	mean	SD	(N)	mean	SD	(N)	mean	SD	(N)	mean	SD	(N)
Need for more instructions (0–4) ^2^	G	2,0		1	2,9	1,4	10	2,1	1,4	8	2,6	0,7	9	3.3*	0,8	7
	TZ	2,1	1,6	8	2,5	1,3	13	3,0	1,0	7	2,6	1,4	12	2.3*	0,7	9
Complexity/multidisciplinarity of activity (0–4) ^2^	G	4,0		1	3,1	1,1	10	2,8	0,9	8	3,2	1,1	9	3,0	0,9	8
	TZ	2,4	1,5	8	2,5	1,0	13	2,9	0,9	7	2,8	1,1	12	2,2	1,0	9
Communication requirements among members during this activity (0–4) ^2^	G	3,0		1	3,3	0,9	10	2.5*	0,8	8	3,6	0,7	9	3,6	0,5	8
	TZ	2,7	1,1	7	3,8	0,6	12	3.5*	0,8	6	3,5	0,7	11	3,1	0,8	8
Degree of stakeholder participation (0–4) ^2^	G	4,0		1	2,6	1,2	10	1.2**	1,0	8	3,1	1,1	9	3,0	0,9	8
	TZ	3,1	1,1	8	3,2	0,9	13	2.8**	0,4	7	3,3	0,8	12	2,8	0,8	9
Percentage of task completed (%)	G	90		1	60	16,3	9	70	14,6	7	49	31,9	8	26	25,8	7
	TZ	92	9,8	7	61	16,8	12	47	40,5	6	5	15,5	12	33	18,7	9
Project member satisfaction during process (0–4) ^2^	G	3,0		1	1,8	1,2	10	2,0	1,2	7	2,1	0,9	9	1,6	0,5	5
	TZ	3,0	0,5	8	2,4	0,7	13	2,0	1,0	7	2,6	0,5	12	2,2	0,7	9
Final project member’s satisfaction after >2 years (0–4) ^2^	G	3,0		1	1.8*	1,1	10	2,0	1,2	7	2,4	1,0	9	1,5	0,8	6
	TZ	3,1	0,7	7	2.7*	0,5	12	2,0	1,1	6	2,7	0,8	12	2,1	0,8	9
Amount of conflicts / tension experienced (0–4) ^2^	G	2,0		1	1,7	1,1	10	0,4	0,7	8	1,6	0,9	9	0,6	0,7	8
	TZ	1,0	0,9	8	1,4	1,2	13	0,3	0,5	7	1,7	1,0	12	1,1	1,1	9

^1^ Stakeholder participation intensity levels: 0: information; 1: consultation; 2: cooperation; 3: collaboration; 4: empowerment (Fig 1)

^2^ ratings: 0: none; 1: low; 2: medium; 3: high; 4: very high

^3^ accumulated No. of days for one step

Significance level

*p < 0.05

**p < 0.01(Mann-Whitney U-test)

### Data background, evaluation process and data analysis

Prior to the evaluation, the assessment criteria and the scale per step were discussed and defined by a core team of Tanzanian and German scientists. The outcome reflected the concepts of [23], who investigated the participation intensity differentiating among information, consultation, cooperation, collaboration and empowerment (decision-making power). As communication was considered pivotal in such a multi-disciplinary setting, emphasis was placed on communication (who, how, where, with whom, and when) and on the communication failures that were experienced. We agreed to have eight assessment parameters scored using either a 5-point Likert scale (0: none; 1: low; 2: medium; 3: high; 4: very high; [24] or an estimated percentage. These eight parameters indicating the quality of participatory action in R&D project collaboration were included in a questionnaire as follows (Tables 1 and 2): *Need for more instructions; complexity/multidisciplinarity of activity; communication requirements among members during this activity; degree of stakeholder participation; percentage of task completed; project member satisfaction during process; final project members’ satisfaction after >2 years; amount of conflict/tension experienced*. Using these parameters, each research step mentioned above was assessed by project members. For instance, a high need for more instructions (parameter 1) in any of the ten research steps was scored with a “3”. The responding members, all of whom were non-anonymous, also provided narratives of critical observations or bottlenecks experienced, as well as recommendations. The assessment task and procedures were shared with only 39 consortium members who were deeply involved with this research. We received 19 assessments from Tanzania and 12 from Germany, including 8 female and 23 male respondents and 16 junior and 15 senior researchers (S1 Table).

Information obtained from the quantitative assessment was statistically analysed with IBM SPSS Statistics 22. We tested a) for significant perception differences according to nationality (German versus Tanzanian), gender (female versus male) and institutional status (senior scientist versus junior scientist (PhD students and postdocs) by using the Mann-Whitney U-test for rating data on a non-parametric scale [24]. Non-significant differences were also indicated if the average scores differed by at least 1.0. We also noted large differences within a particular group, such as Tanzanian members, as expressed by a high standard deviation (SD). We tested b) for correlations of nationality, gender and institutional status with specific project steps and perception parameters (Pearson product-moment correlation). The qualitative feedback narratives received from 11 Tanzanian members and 10 German members were systematically evaluated and cited, if needed, to support the assessments. Hence, this constitutes a mixed methods approach (both quantitative and qualitative) in data generation, with the aim of achieving representativeness among project participants and at the same time greater analytical accuracy of their behaviour and perceptions towards this participatory research project [25].

## Results

### Guidance on the research process and steps, as provided by coordinators and team leaders

The guidance and instructions provided by the coordinators varied largely, as did the number of involved scientists and stakeholders per analytical step (Tables 1 and 2, S1 Table). Some steps were particularly complex, requiring the involvement of a larger number of scientists and stakeholders, as well as more detailed instructions. This was true for steps four (Identifying 3–5 UPS per FVC component), seven (UPS implementation, testing, adaptation), and nine (UPS monitoring & impact assessment). These steps were also consistently linked to higher levels of stakeholder participation intensity and to distinctly higher cooperation among scientists. Although these complex activities demanded and were given more time by the project coordinators (Tables 1 and 2), they were regularly followed by significant delays before the activity was finalized. Moreover, they triggered particularly high numbers of reminders (up to 20) from project leaders before they were finalized. Higher numbers of involved stakeholders did not necessarily require a higher degree of cooperation among scientists, nor did they lead to increased delays.

### North-South (culture)-specific assessments

#### Perceptions of the participatory process

Consortium members’ perceptions indicated that the quality of the participatory project process largely differed between the research activities and the eight parameters evaluated; however, the comparison of Tanzanian versus German assessments provided a more differentiated picture (Tables 1 and 2).

Need for more instructions: The more complex activities—for instance, research steps (2), (4), (9), and (10)—were indicated as requiring more instructions. Tanzanians expressed a higher need for instructions on research activities in steps (3) and (8) but significantly (p < 0.05) lower needs in step (10) compared to their German counterparts. In general, the involvement of high numbers of project teams and members triggered more demand for instructions and produced activity delays. High rating disunities (high standard deviations, SD) among German members were found, for instance, for steps (4), (7), and (8), implying that these partners were unequally informed or involved. A German member found that “Identifying the UPS [steps 4 and 5] was a challenge … due to the lack of instructions between the scientific partners in Germany and the local [Tanzanian] members responsible for completing the task.” A Tanzanian member, however, stated that “this [step 4 and 5] was not difficult as the foundation was already set from other earlier processes… and the prioritization process was participatory.”

Complexity/multidisciplinarity of activity: All activities except (1) and (10) were given high to very high ratings (2.5–4.0) for complexity/multidisciplinarity, with particularly high ratings by Germans. This indicates the multiple challenges this type of participatory research presented to the project members, especially those less familiar with the local settings and local participation. A Tanzanian member stated that “the project is very complex, it its multidisciplinary; therefore, there are some tensions, especially in final decisions and [UPS] implementations.” Communication requirements among members during this activity: The communication requirement for all activities was perceived to be medium to very high (2.9–3.8), with higher ratings by Tanzanians in most cases. This is particularly true for step (8), with significant differences (p<0.05) between both nationalities. This finding reflects the need for repeated information exchange via different communication pathways between both nationalities; this need was more distinctly expressed by Tanzanians. A German member expressed the challenge that “different expectations, different institutional and personal agendas, different levels of professionalism, and often different definitions of the same terms rendered the communication between the scientific members of the consortium very difficult. …”. Particularly high ratings were received for activities involving many project members. Interestingly, respondents of both nationalities agreed quite highly among themselves (low SD).

Degree of stakeholder participation: Ratings on degree of stakeholder participation differed largely among activities, ranging from low to very high (1.2–4.0). Tanzanians tended to indicate higher participation rates, particularly for steps (4) and (8), with significant differences (p < 0.05 and p < 0.001, respectively) between Tanzanians and Germans. Low ratings also reflected the lower number of stakeholders involved. Maximum degrees of stakeholder participation were reported for those activities where few (80–200) stakeholders were involved but more interaction took place.

Percentage of task completed at the time of the survey: The assessments for this parameter differed significantly, ranging from 26% to 100% depending on the type and schedule of activities. While some activities were still on-going, such as (7), (8), (9), and (10), other activities, such as (1), were not fully completed and required finalization. Interestingly, we found high disunity among project members, especially Tanzanians, in steps (1), (4), (8) and (10), indicating that they disagreed about the task status reached.

#### Perception of satisfaction and tensions experienced

Project members’ satisfaction during activity: The ratings for project members’ satisfaction during the activity ranged from low to high (1.0–3.3), always with lower or equal satisfaction rates among German members and even significantly lower satisfaction (p < 0.05) for step (4). Higher satisfaction among both nationalities was linked with higher degrees of stakeholder participation, for instance with activities (3), (5) and (6). Lower satisfaction among both nationalities was observed for activities such as (8) and (10), which demanded more time and desk work.

Final project members’ satisfaction after 1–2 years: Ratings for this parameter differed greatly, ranging from low to very high (1.0–3.7), with regularly lower or equal satisfaction rates among German members and significantly lower satisfaction (p < 0.05) for step (7). The final satisfaction ratings differed only slightly from those for members’ satisfaction during the activity. This finding indicates that despite lower satisfaction during an activity, this could not be resolved during the activity’s finalization, as one German member stated: “strong interventions from the German side kind of saved the process, but I think this [step 4] was the weakest point”. There was high overall agreement on satisfaction ratings among the respondents.

Amount of conflict/tension experienced: The number of conflicts experienced was perceived to be zero to medium (0.2–2.0), except for one person who indicated very high tension at step (3). In most cases, the German counterparts experienced more tension than their Tanzanian colleagues. Most conflicts were perceived during steps (4), (7), and (9); these were considered complex activities requiring a high degree of cooperation among project members, a high need for more instructions, high communication requirements, and a high degree of project member and stakeholder participation. They also required many reminders before finalizing the activity. Altogether, “conflicts were lived in a forward thinking and constructive way that helped foster mutual understanding and respect”, as a German member stated.

### Gender-specific assessments

The respondents’ perceptions of the quality of the participatory process were analysed for gender-specific differences (Table 3). With only eight females compared to 23 male respondents, the female N was small in many cases. Nevertheless, for step (4), with an overall high N of researchers, it was found that females perceived the degree of stakeholder participation to be significantly higher (p < 0.05) compared to their male counterparts. Additionally, for step (7), they indicated significantly higher accordance (p < 0.05) with the status of the task reached by that point. Likewise, both parameters correlated highly with the gender making the assessment (Table 4). In general, female scientists tended to be more satisfied with the participatory process, with one Tanzanian female member stating “…levels of tension were minimal, as everything was conducted in transparency, channels of communication were very clear for each task.”

**Table 3:** Significance of differences in gender and status across participative steps and different assessment parameters (S1 Table).

	Step1	Step2	Step3	Step4	Step5	Step6	Step7	Step8	Step9	Step10
Gender	-	-	-	P4:	-	-	P5:	-	-	-
				2,8(m)*			56.9(m)*			
				1,0 (f)*			86.6 (f)*			
Status	-	-	-	-	P1:	-	P4:	-	-	-
					3.2 (j)*		3.6 (j)**			
					1.3 (s)*		2.4 (s)**			

male (m) versus female (f); junior scientist (j) versus senior scientist (s); assessment parameters: P1: Need for more instructions; P2: Complexity/multidisciplinarity of activity; P3: Communication requirements among members during this activity; P4: Degree of stakeholder participation; P5: Percentage of task completed; P6: Project member satisfaction during process; P7: Final project member’s satisfaction after >2 years; P8: Amount of conflicts / tension experienced. significance level:

*p < 0.05

**p < 0.01; (Mann-Whitney U-test)

**Table 4:** Pearson correlations of nationality, gender and institutional status across participative steps and different assessment parameters (S1 Table).

	Step1	Step2	Step3	Step4	Step5	Step6	Step7	Step8	Step9	Step10
Nationality	P8*	P5**, P6***, P7*,P8*	-	P4*, P6**	-	-	P7*	P3*, P4**		P1*
Gender	-	-	-	P4*	-	-	P5***	-	-	-
Status	-	P2*	-	-	P1*, P7*	-	-	-	-	-

assessment parameters: P1: Need for more instructions; P2: Complexity/multidisciplinarity of activity; P3: Communication requirements among members during this activity; P4: Degree of stakeholder participation; P5: Percentage of task completed; P6: Project member satisfaction during process; P7: Final project member’s satisfaction after >2 years; P8: Amount of conflicts / tension experienced. significance level:

*p < 0.05

**p < 0.01

***p < 0.001

### Institutional status-specific assessments

The statistical analysis yielded institutional status-specific or hierarchy-specific differences in perceptions of the participatory process (Table 3). Junior scientists felt significantly more (p < 0.05) need for instructions in step (5) and, interestingly, they perceived higher stakeholder participation in step (7) (p < 0.01) compared to their senior counterparts, likely because they were more involved than their senior colleagues. In step 2, perceptions of activity complexity were also correlated with hierarchical status (Table 4); likewise, in step 5, the assessments of both need for instructions and final members’ satisfaction after 1–2 years correlated with hierarchical status. Hence, hierarchical status in some cases led to specific perceptions of the participatory activities, which we assume are associated with status-specific project activities and/or the scientist’s working experience.

## Discussion

This study aimed to discriminate among different perceptions of PAR according to culture, gender, and institutional status in the context of a large multidisciplinary and multi-cultural project. Fifteen parameters that were previously agreed upon (Tables 1 and 2) were assumed to depict most of the characteristics, complexities, and perceptions experienced [4,5,15]. Of the 39 project members involved with the PAR activities, a total of 31 assessed the eight previously agreed-upon parameters across ten different PAR steps. The N in most cases was statistically sufficient to derive conclusions about the influence of culture, gender, and institutional status on perceptions of the study’s PAR. While the perceptions of scientists differed greatly among different PAR steps, comparing Tanzanian versus German assessments (Tables 1–4) produced a number of interesting insights regarding culture-specific perceptions. These differences in perception are considered important in the context of north-south cooperation and research [12,25,26].

Tanzanian members gave higher ratings to a) the need for instructions, b) communication requirements among members, c) degree of stakeholder participation, d) satisfaction during an activity, and e) final satisfaction after 1–2 years. German project members gave higher assessments of a) the steps’ complexity/multidisciplinarity, thus indicating the multiple challenges of PAR for those less familiar with the local settings, and b) the amount of conflict/tension experienced. These differences indicate culture-specific differences in leadership style, communication, and team relations [10,15] These findings concur well with [26], indicating differences in power distance, individualism, uncertainty avoidance, and long term orientation between both cultures.

In general, higher rating disunities were found among German members compared to their Tanzanian counterparts (Tables 1–3). The peaks of instruction needs, communication requirements and degrees of stakeholder participation indicated by Tanzanians tended to coincide with higher dissatisfaction levels and tensions experienced among German colleagues who were trying to avoid uncertainty (Tables 1–4). This led to tension, particularly across the different cultures [14,26,27]. Due to the nature and requirements of this type of PAR, including knowledge of the local language, culture, formality, and trust [28], as well as low masculinity (solidarity, well-being, support) [26], Tanzanian scientists were more strongly involved in on-the-ground activities than were their German counterparts, who often acted as virtual team members with less connection to field research [29]. Many PAR steps required higher levels of stakeholder participation and local knowledge integration [Fig 1; 7,23] and were regularly organized and performed by experienced Tanzanian team members, with German members waiting for the results to be generated by their colleagues. These activities required more scientist-and-stakeholder and north-south communication and commitments than previously expected [8,10,14].

Narratives by the project’s scientists indicated that in some cases, needs for interdisciplinary north-south collaboration and communication among scientists and local stakeholders were not met by coordinators, task leaders or other members (Tables 1 and 2). This lack of communication and collaboration affected overall communication and team relations, particularly due to the mainly virtual form of collaboration used in the project [15]. A German member found that “overall, [North-South] communication was the most difficult task among the project [scientists]…as a result, communication with the stakeholders similarly led to confusing results.” Our findings also coincided with [21], indicating that multicultural project collaboration was smoother if leaders showed an awareness of cultural variation; these findings thus indicate the challenge of creating “effective cross cultural collectivism, trust, communication and empathy in leadership” [10]. Levels of participation intensity, as indicated in Fig 1, and methods of participation [30,31] during the PAR steps that involved both German and Tanzanian scientists and local stakeholders had not been fully defined and communicated previously, as suggested by [32]. The levels of cooperation, empowerment and participative collaboration were obviously underestimated by most project members at the outset and were still assessed as significantly different between nationalities during this investigation (Tables 1 and 2). This resulted in the need for post hoc definitions, discussions and meta-communication [33]. Our results clearly show that cultural diversity and culture-specific conflict-triggering factors played a role in triggering dissatisfaction. As a Tanzanian member stated, “some partners dominated others to the point that some actions were imposed instead of using a consensus building and negotiation approach”. The literature shows that different communication types, behaviours and expectations are known to trigger conflicts [10,21,27] that, in turn, require different conflict-management approaches [34–36]. Higher dissatisfaction, however, ultimately translates into poorer communication and less effective transformation of newly gained knowledge into action [11], as was also experienced in a few north-south communication breakdowns during the first two project years. In the third and fourth years, the increased competence and awareness of multicultural and contextual factors helped the project and team leaders’ to establish relationships, communicate and approach challenges more effectively, as also indicated by [29].

Hierarchical status in institutions and in the research project, as well as gender, were shown to significantly affect perceptions of this PAR process [5,12,20,26,27,37] and correlated with specific assessment parameters (Tables 3 and 4). For instance, female scientists tended to be more satisfied with the PAR, while hierarchical status in some cases led to differing perceptions of the PAR activities, which was assumed to be associated with status-specific involvements in specific PAR activities and/or the scientists’ personal experience and perceptions changing depending on their place in the hierarchy [28,38]. A major cultural difference was the difference in power distance between Tanzanian and German members, defined by [26] as “the extent to which the less powerful members of institutions … within a country expect and accept that power is distributed unequally”. In Tanzania, the power distance calculated by [26] was twice as high compared to Germany, and Tanzanian members’ behaviour towards an institutional hierarchy was more respectful [25].

In this particular study, the perceptions of many PAR parameters differed significantly depending on nationality, hierarchical status in one’s institution and the research project, and gender. While it is assumed that for any other large multi-disciplinary cross-cultural PAR project, the assessments would be somewhat different, the perceptions are still expected to differ between nationalities, hierarchies and gender, as supported by the literature [5,12,15,25–27,36,38]. The reasons behind these different perceptions cannot be entirely identified, but we suggest that historically driven cultural values, societal rules and hierarchies [25–27], resulting education, and professional environments require special consideration in the project design of north-south collaborations.

## Conclusions and recommendations

In conducting an evaluation of scientists’ perceptions of a large multi-disciplinary cross-cultural PAR project focused on a rural FVC in Tanzania, we found that the PAR activities required higher levels of stakeholder participation, more north-south cooperation and communication among members, and more instruction input from project management than previously expected. Underestimating these parameters was likely to trigger tension and dissatisfaction within and between different cultures. Perceptions significantly differed depending on nationality, hierarchical status in institutions and the research project, and gender. Therefore, these differences require particular consideration at the outset of a project and during the implementation of this type of cooperation. Differing perceptions in this multi-disciplinary, cross-cultural PAR also required more transparent and earlier communication between all partners and institutional levels involved. We recommend that communication be facilitated by regular in-house meta-communication and conflict-management training. We stress the fundamental importance of bidirectional continuous communication pathways. The formation of consortia that include both southern and northern institutions and colleagues as more equal partners may help with the transition from a one-directional transfer of capacity from north to south into a more equal partnership characterized by mutual learning. Especially at the beginning of such projects, it is crucial to preview the budget and to set aside time for sensitization regarding crucial areas of north-south understanding and communication in order to level out asymmetric knowledge and related tensions.

There is still insufficient scientific research into how large north-south PAR cooperation can be enhanced. We suggest that further research should investigate and evaluate the various possible combinations of large multi-disciplinary, multi–institutional, and multi-cultural PAR projects. Adding to PAR theory and practice this could help identifying specific mechanisms and activities required in such cultural, disciplinary and geographical context.

## Supporting information

S1 Table Data set on perception assessments across participatory research actions. (ZIP)
